# Factors associated with *loss to follow-up* before and after treatment initiation among patients with tuberculosis: A 5-year observation in China

**DOI:** 10.3389/fmed.2023.1136094

**Published:** 2023-04-25

**Authors:** Youli Jiang, Jingfang Chen, Meng Ying, Linlin Liu, Min Li, Shuihua Lu, Zhihuan Li, Peize Zhang, Qingyao Xie, Xuhui Liu, Hongzhou Lu

**Affiliations:** ^1^Hengyang Medical School, School of Nursing, University of South China, Hengyang, China; ^2^Shenzhen Third People’s Hospital, Shenzhen, China; ^3^Department of Intelligent Security Laboratory, Shenzhen Tsinghua University Research Institute, Shenzhen, China

**Keywords:** tuberculosis, loss to follow-up, risk factor, observation, insurance

## Abstract

**Background:**

*Loss to follow-up* (LTFU) is a significant barrier to the completion of anti-tuberculosis (TB) treatment and a major predictor of TB-associated deaths. Currently, research on LTFU-related factors in China is both scarce and inconsistent.

**Methods:**

We collected information from the TB observation database of the National Clinical Research Center for Infectious Diseases. The data of all patients who were documented as LTFU were assessed retrospectively and compared with those of patients who were not LTFU. Descriptive epidemiology and multivariable logistic regression analyses were conducted to identify the factors associated with LTFU.

**Results:**

A total of 24,265 TB patients were included in the analysis. Of them, 3,046 were categorized as LTFU, including 678 who were lost before treatment initiation and 2,368 who were lost afterwards. The previous history of TB was independently associated with LTFU before treatment initiation. Having medical insurance, chronic hepatitis or cirrhosis, and providing an alternative contact were independent predictive factors for LTFU after treatment initiation.

**Conclusion:**

Loss to follow-up is frequent in the management of patients with TB and can be predicted using patients’ treatment history, clinical characteristics, and socioeconomic factors. Our research illustrates the importance of early assessment and intervention after diagnosis. Targeted measures can improve patient engagement and ultimately treatment adherence, leading to better health outcomes and disease control.

## Introduction

Globally, the estimated number of deaths from tuberculosis (TB) increased from 1.4 million to 1.6 million between 2019 and 2021 (1). *Loss to follow-up* (LTFU) remains a leading cause of these deaths (2). According to the World Health Organization’s (WHO) global TB report of 2021, the proportion of LTFU has persisted at 6% globally from 2012 to 2019, with a substantial number of LTFU cases in recent years (2) resulting in treatment failure or death, disease transmission in households and communities, and drug resistance (3–5).

China has the second-highest tuberculosis (TB) disease burden in the world. According to the latest data from the Chinese Center for Disease Control and Prevention, 842,000 new cases of TB were reported in 2020, and the incidence rate of TB in China was 59 per 100,000 population (4). There are several major challenges restricting the decline of TB incidence, including the high proportion of patients who are lost to follow-up (LTFU), nationwide socioeconomic imbalance, high prevalence of latent TB infection, etc. In China, the proportion of LTFU was reported to be 28.2% (6). In a meta-analysis, LTFU before treatment initiation accounted for 18% of all LTFU cases in Africa and 13% in Asia (China was not included) (7). During treatment, the rate of LTFU was reported to be 15% in France (8, 9). The findings from previous studies suggest that various factors may contribute to LTFU among TB patients. A previous study analyzed LTFU before treatment initiation and identified previous anti-TB treatment without a recorded phone number as independent risk factors for pre-treatment LTFU (8, 9). Current studies report LTFU after treatment initiation to be associated with being male (10), being part of a migrant population (11), having multi-resistance to anti-TB drugs (10, 12) and suffering treatment side effects (13). These influential factors may vary according to domestic TB management policy and socioeconomic changes as well as the patient’s clinical status. However, there is no conclusive evidence on the factors related to LTFU in China, and the available studies in the country are scarce and inconsistent. Therefore, we aimed to investigate the factors associated with LTFU before and after the initiation of TB treatment using long-term monitoring data.

## Methods

### Study design and participants

This retrospective cross-sectional study was conducted in Shenzhen, China. Shenzhen is a major gateway city in China with a high population density and a large migrant population, making it a potential hotspot for TB transmission and a challenge for TB control efforts. The incidence of TB in Shenzhen was reported 50.13/100,000, similar with the pooled incidence of China (5). We compared LTFU cases with those recorded as “cured,” “treatment success,” or “treatment completed” among patients with TB who were registered on the Tuberculosis Information Management System (TBIMS) between January 1, 2017, and December 31, 2021. The TBIMS is a nationwide TB monitoring system affiliated to the National Clinical Research Center for Infectious Diseases (Shenzhen) that collects key information on TB cases reported *via* the country’s TB care facility network. More than 87% of the tuberculosis patients included in our study were from areas outside of Shenzhen, and 60% of the patients were from provinces and regions other than Guangdong, mainly including 33 provinces and regions such as Hunan, Hubei, Sichuan, Chongqing, Jiangxi, and Henan. This could be due to various reasons such as migration for work, study, or other reasons. It is important to understand the extent and patterns of migration to Shenzhen and other cities to develop effective strategies for tuberculosis control and prevention.

The inclusion criteria followed the standards of the WHO (14): (1) A bacteriologically confirmed TB case had a positive biological specimen confirmed by smear microscopy, culture, or WHO-approved rapid diagnostics (such as GeneXpert MTB/RIF). (2) A clinically diagnosed TB case did not fulfill the criteria for bacteriological confirmation but was diagnosed as active TB by a clinician or other medical practitioner who agreed to provide the patient with a full course of TB treatment. The exclusion criteria of the patients were as follows: (1) patients who were undergoing anti-TB treatment according to a medical plan, (2) patients recorded as “dead,” with “treatment failure,” or who were “not evaluated,” and (3) patients with incomplete clinical data. This study was approved by the ethics committee (2022–027). The hospital is committed to using these statistics without disclosing any of the patients’ personal information, and it complies with the Declaration of Helsinki regarding confidentiality and ethical standards.

### Key definition

According to the WHO’s *Definitions and Reporting Framework for Tuberculosis – 2013 Revision*, which was updated in 2021 (15), LTFU in patients with TB is defined as “a TB patient who did not start treatment or whose treatment was interrupted for two consecutive months or longer.”

The definitions for Cured: A patient who was initially bacteriologically positive for TB and who was then bacteriologically negative in the last month of treatment and at least one previous occasion. Treatment success: A patient who was initially bacteriologically positive for TB and who was then either cured or completed treatment. Treatment completed: A patient who completed treatment without evidence of failure but without a bacteriological result in the last month of treatment (16).

Individuals who are not registered residents of Shenzhen or have not lived in Shenzhen for more than 6 months are considered as migrant population.” (17).

### Data collection

Patient data were collected from the TBIMS and the hospital information system (HIS). The TBIMS and HIS are two independent registration systems of different scales, and the patients’ information retrieved from these two systems cannot be matched automatically. Therefore, to consolidate and analyze the data, we extracted data from the two systems and matched them with four consistent variables: name, sex, inpatient ID, and outpatient ID. 26,695 TB patients retrieved from TBIMS and HIS were matched. The process was monitored by two researchers to ensure data quality and completeness (QX and YJ).

We identified the independent variables that matched the pre-defined risk factors associated with LTFU in previous studies and other possible factors that have not been reported to date. The variables included in this study were sex, age, marital status, employment status, mobility population, provision of an alternative contact, medical insurance, underlying medical conditions, concurrent pulmonary and extrapulmonary TB, drug-resistant (DR) TB/rifampicin-resistant (RR) TB, negative results of acid-fast bacilli (AFB) smear/GeneXpert/culture, previous history of TB, had ever been hospitalized for TB, admission to emergency department, and occurrence of adverse drug reactions.

### Statistical analysis

To determine the occurrence of LTFU, we stratified all participants’ inpatient and outpatient follow-up records into three categories based on treatment outcomes: non-LTFU (treatment success or referrals), LTFU before treatment initiation, and LTFU after treatment initiation. First, patients with successful treatment were identified according to their treatment records and were further screened on the TBIMS for referral, updated residency, and follow-up status to exclude migrant populations who returned to their home cities for treatment. Finally, according to the occurrence time of LTFU, all the LTFU cases were divided into two groups (pre-treatment and during treatment) and were analyzed, respectively, for dropout time and risk factors.

The processing and statistical analysis of the data were completed using Python 3.9 and Statistical Package for Social Science (SPSS) version 25.0 software. Continuous variables were expressed as median (interquartile range [IQR]) or mean ± standard deviation ( $\bar{X}$ ± s). Based on the results of a normality test, continuous variables were evaluated using a *t* test or a Mann–Whitney *U* test. Categorical variables were described in frequencies and percentages and were assessed using an χ^2^ test. The Kaplan–Meier method was used to plot survival curves and estimate the unadjusted time to LTFU across the treatment period. A binary stepwise forward logistic regression analysis was utilized to identify the independent influencing factors of LTFU. To control confounding factors, the results of the univariate analysis were presented in terms of adjusted odds ratio (aOR).

## Results

**Figure 1:**
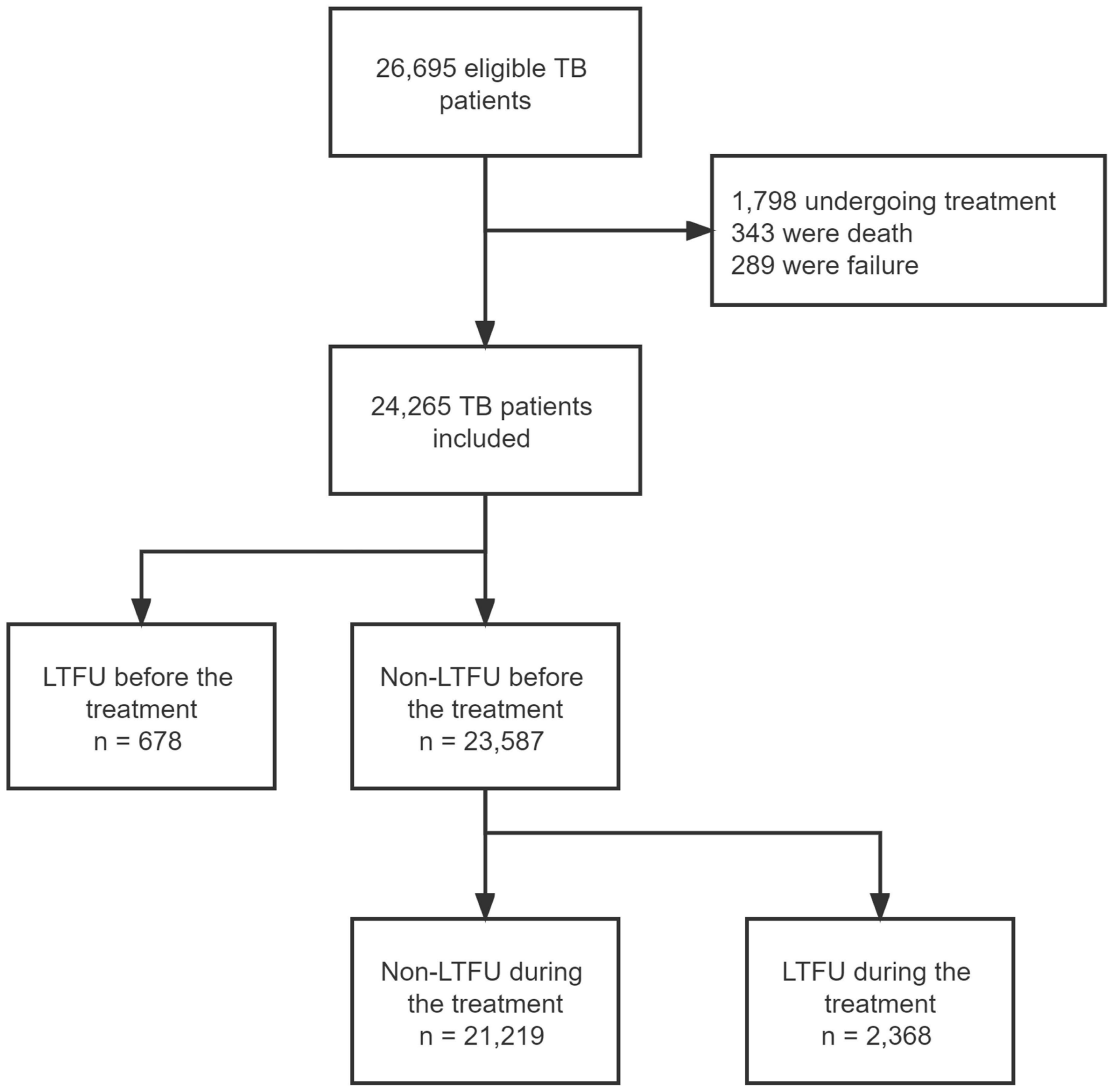
Flow diagram of the study.

Between January 2017 and December 2021, we collected 26,695 eligible individuals who were diagnosed with TB-related diseases. Patients with a record of treatment (*n* = 1,798), death (*n* = 343), and failure (*n* = 289) were excluded. Finally, 24,265 patients were included in our analysis. A total of 3,046 (12.55%) patients were recorded as LTFU, including 678 patients who were unable to start anti-TB treatment and 2,368 who were LTFU during the treatment regimen (Figure 1). Among all the patients with TB, 15,889 (65.5%) were male, and with a similar median age to that of the all LTFU cases (median age: 37 years, interquartile range [IQR]: 27–53). The proportion of employed/self-employed patients who were LTFU before and after the initiation of treatment was 54 and 52%, respectively. The detailed characteristics and information of the patients in our dataset are presented in Table 1.

**Table 1:** Sociodemographic characteristics and clinical information of loss-to-follow-up (LTFU) rate in patients with tuberculosis (TB) vs. patients adhering to treatment from 2017 to 2021 (*n* = 24,265).

Variables	Total *n* = 24,265 (%)	Before treatment initiation		*p*-value	After treatment initiation		*p*-value	Quantile regression analysis: median (IQR) time to LFU (months)
		Non-LTFU *n =* 23,587 (%)	Pre-treatment LTFU *n =* 678 (%)		Non-LTFU *n* = 21,219 (%)	During treatment LTFU *n =* 2,368 (%)		
Male	15,889 (65.5)	15,444 (65.5)	445 (66.5)	0.568	13,913 (65.6)	1,529 (64.4)	0.234	2 (1–4)
Age (Median [IQR])	37 (27–54)	37 (27–54)	37 (27–53)	0.502	37 (27–54)	37 (27–53)	0.248	2 (1–4)
Married	16,669 (68.7)	16,217 (68.7)	452 (67.6)	0.522	14,595 (68.8)	1,622 (68.2)	0.609	2 (1–4)
Employed/self-employed	10,398 (42.9)	10,079 (50.0)	319 (54.8)	0.022	9,018 (49.7)	1,061 (52.5)	0.018	2 (1–4)
Migrant	14,828 (61.1)	14,416 (61.1)	412 (61.6)	0.798	12,999 (61.3)	1,417 (59.6)	0.124	2 (1–4)
Medical insurance	17,285 (71.2)	16,836 (71.4)	449 (67.1)	0.017	15,257 (71.9)	1,579 (66.5)	0.000	3 (1–5)
Diabetes mellitus	1,792 (7.4)	1,731 (7.3)	61 (9.1)	0.082	1,540 (7.3)	191 (8.0)	0.166	2 (1–4)
chronic hepatitis or cirrhosis	1,395 (5.7)	1,350 (5.7)	45 (6.7)	0.271	1,185 (5.6)	165 (6.9)	0.007	2 (1–4)
Concurrent pulmonary & extrapulmonary TB	3,296 (13.6)	3,198 (13.6)	98 (14.6)	0.415	2,856 (13.5)	342 (14.4)	0.207	2 (1–4)
RR/DR-TB	729 (3.0)	709 (3.0)	20 (3.0)	0.982	646 (3.0)	63 (2.7)	0.288	3 (1–5)
Negative results of AFB smear/GeneXpert/Culture	11,335 (46.7)	10,907 (46.2)	428 (64.0)	0.000	9,326 (43.9)	1,581 (66.5)	0.000	2 (1–4)
Previous history of TB	1,209 (5.0)	1,163 (4.9)	46 (6.9)	0.022	1,042 (4.9)	121 (5.1)	0.697	2 (0–4)
Had been hospitalized	15,415 (63.5)	15,284 (64.8)	131 (13.1)	0.000	14,809 (69.8)	475 (20.0)	0.000	3 (1–5)
Provision of an alternative contact	4,194 (17.3)	19,525 (82.7)	546 (81.6)	0.445	17,611 (83.0)	1,914 (80.6)	0.003	2 (1–4)
Consultation in hospital				0.000			0.000	
Emergency	15,067 (62.1)	14,909 (63.2)	158 (23.6)		14,284 (67.3)	625 (26.3)		2 (1–4)
Outpatient	9,198 (37.9)	8,687 (36.8)	511 (76.4)		6,936 (32.7)	1,751 (73.7)		2 (1–4)
Occurrence of Adverse drug reaction	1,444 (6.0)	\	\		1,222 (5.8)	183 (7.7)	0.000	2 (1–3)

### Differences in cases who lost to follow-up before and after the initiation of treatment

Table 2 shows that there was a statistical difference between patients with a TB history before treatment initiation and after (*p* = 0.048). In the patients, 81.9% of individuals who had LTFU before treatment initiation were consulted in an outpatient clinic; the difference between before and after the initiation was statistically significant, with a value of *p* = 0.001 among those who visited an outpatient clinic.

**Table 2:** Difference in characteristics of loss to follow-up (LTFU) between pre-treatment and during tuberculosis (TB) treatment in patients with a previous history of TB (*n* = 167).

Variables	Patients with a previous history of TB		*p*-value
	Pre-treatment LTFU *n* = 46 (%)	During treatment LFTU *n* = 121 (%)	
Male	29 (63.0)	74 (61.2)	0.823
Age	45 (34–59)	43 (29–55)	0.553
Medical insurance	34 (73.9)	70 (57.9)	0.056
Concurrent pulmonary & extrapulmonary TB	8 (17.4)	34 (28.1)	0.154
Negative results of AFB smear/GeneXpert/Culture	26 (56.5)	80 (66.1)	0.250
Had been hospitalized	6 (13.0)	32 (26.4)	0.065
Consultation as outpatient	41 (89.1)	76 (62.8)	0.001

All the variables in Table 1 were used to compare the difference between before and after treatment initiation. We found a statistical difference between the proportion of patients who had previously been treated for TB in these two phases (*p* = 0.048).

### Occurrence time of loss to follow-up

**Figure 2:**
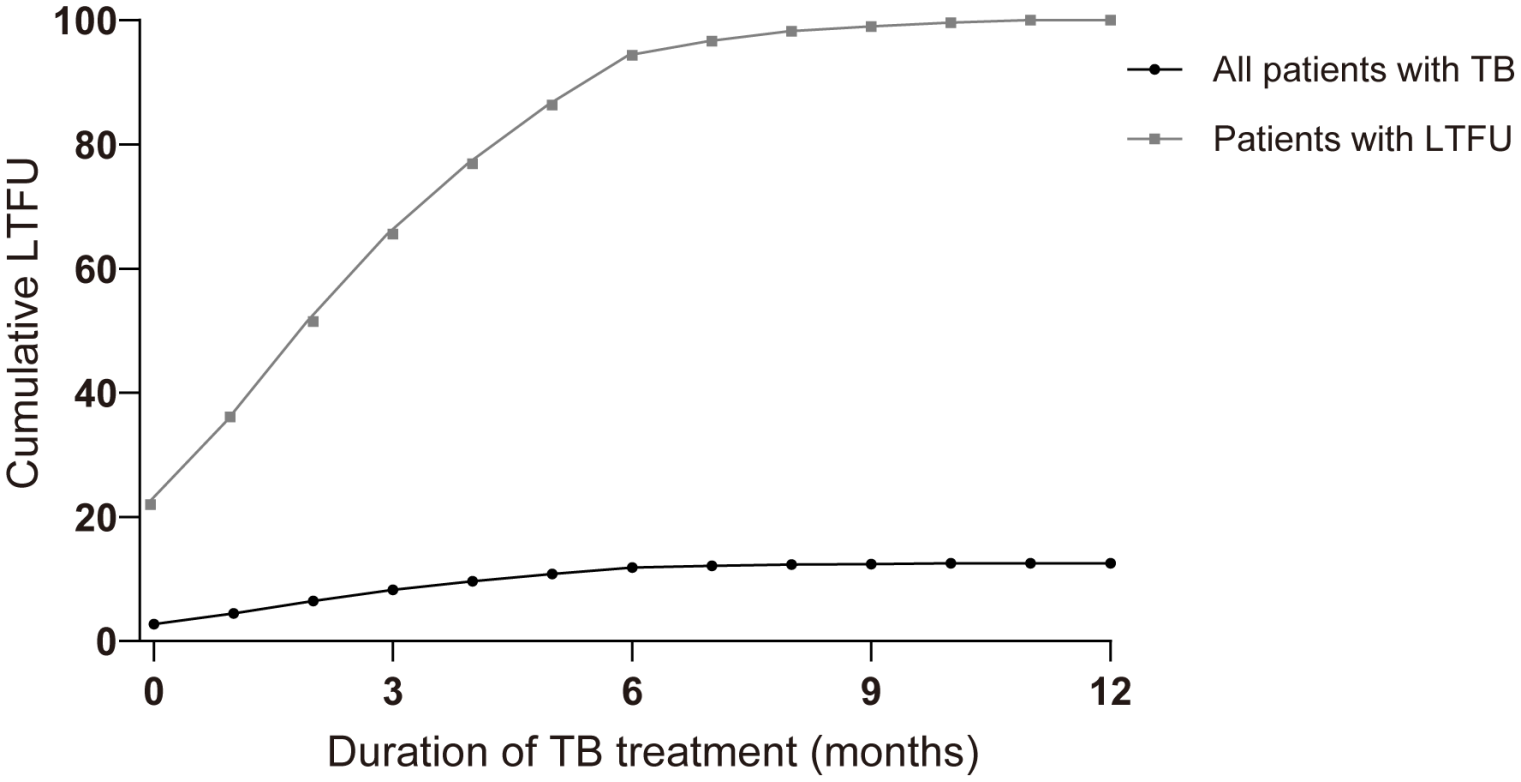
Comparison of cumulative percentage and event occurrence time of loss-to-follow-up (LTFU) cases in all patients with tuberculosis (TB) and patients with LTFU only over the treatment course (months). Nb. All patients with TB (*n* = 24,265), patients with LTFU (*n* = 3,046).

Of all the LTFU cases, 21.98% occurred before treatment initiation. A total of 35.58% of cases occurred within the first month, and more than half had been lost 2 months after the initiation of treatment. The accumulated LTFU rate reached 66.90% (*n* = 1963) by 4 months and 93.74% by 6 months (Figure 2).

**Figure 3:**
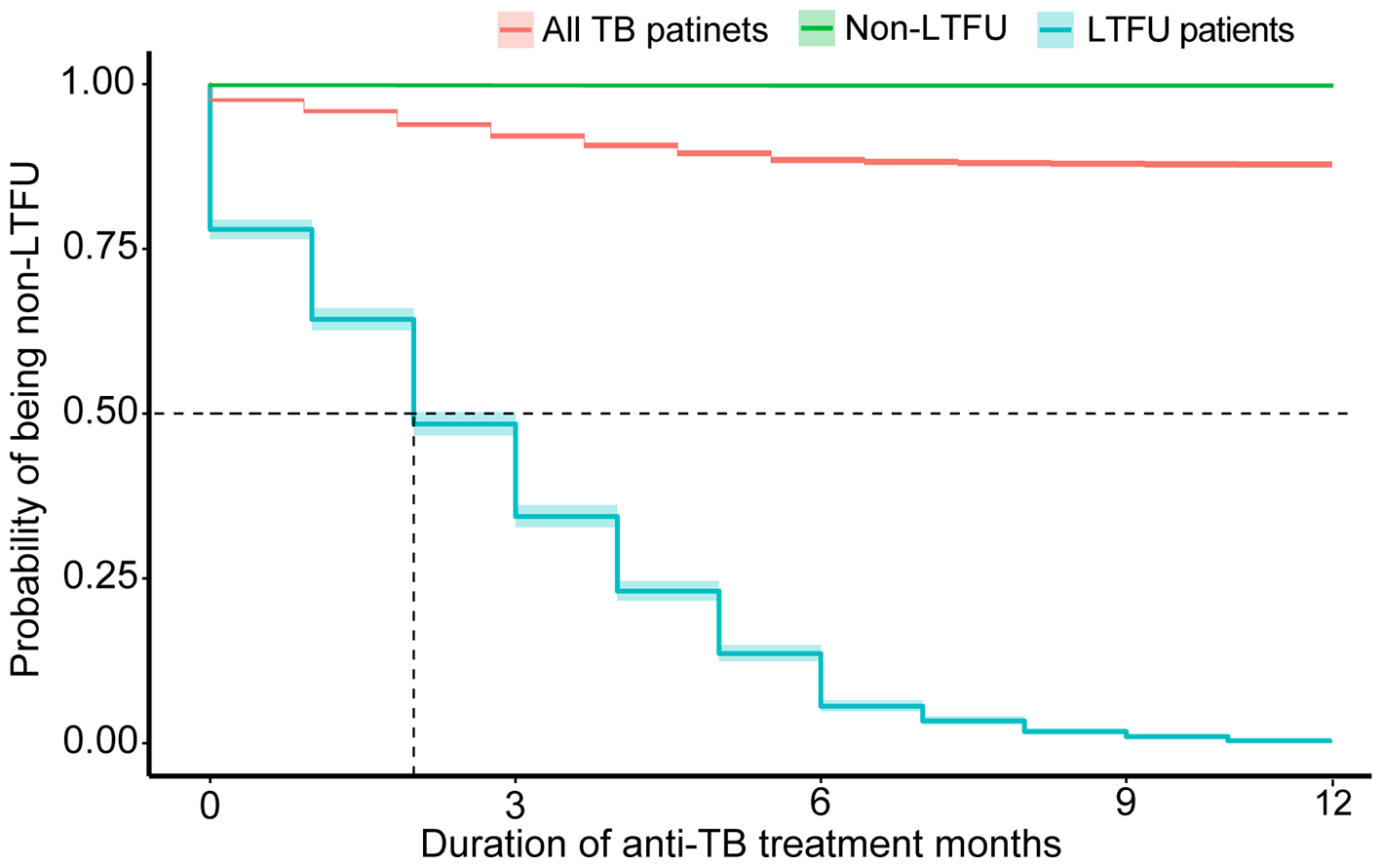
Time to loss to follow-up after diagnosing TB, using Kaplan–Meier analysis (*n* = 24,265).

In Table 1, the median time (IQR) to LTFU occurrence for all patients was 2 months (1–4 months). A further analysis of additional variables revealed a broadly similar median time to that of LTFU. The incidence of LTFU was higher during the initial phase of treatment (the first 2 months) than during the continuation phase, and the curve leveled off after 6 months of treatment (Figures 2, 3).

### Risk factors associated with LTFU before and after TB treatment initiation

We used univariate and multivariate logistic regression models to identify the risk factors that may have predisposed the participants to LTFU. A value of *p* < 0.05 denoted statistical significance. As shown in Table 1, the following risk factors were found to be associated with LTFU: employed/self-employed, medical insurance, chronic hepatitis or cirrhosis, negative AFB smear/GeneXpert/culture results, previous history of TB, had ever been hospitalized for TB, provision of an alternative contact, occurrence of adverse drug reaction, and admission to emergency department (Table 3).

**Table 3:** The adjusted odds ratio of factors associated with loss to follow-up (LTFU) before and after treatment initiation among patients with tuberculosis (*n* = 24,265).

Variables	Category	LTFU before the initiation		LTFU after the initiation	
		Adjusted odds ratio (95% CI)	*p*-value (*α* = 0.05)	Adjusted odds ratio (95% CI)	*p*-value (*α* = 0.05)
Has been hospitalized	Yes (vs No)	0.22 (0.18–0.28)	0.000	0.19 (0.17–0.22)	0.000
Consultation at hospital	Emergency (vs Outpatient)	0.38 (0.31–0.47)	0.000	0.40 (0.35–0.44)	0.000
Previous history of TB	Yes (vs No)	1.50 (1.09–2.07)	0.014		
chronic hepatitis or cirrhosis	Yes (vs No)			1.24 (1.02–1.51)	0.031
Occurrence of Adverse drug reaction	Yes (vs No)			1.26 (1.03–1.52)	0.021
Provision of an alternative contact	Yes (vs No)			0.82 (0.72–0.94)	0.003
Medical insurance	Yes (vs No)			0.76 (0.69–0.85)	0.000
Negative results of AFB smear/GeneXpert/Culture	Yes (vs No)	1.45 (1.22–1.73)	0.000	1.75 (1.58–1.94)	0.000
Employed/self-employed	Yes (vs Unemployed/retired)	1.21 (1.02–1.43)	0.029	1.15 (1.04–1.27)	0.005

The results of the multivariate stepwise regression analysis indicated that negative AFB smear/GeneXpert/culture results (aOR = 1.45, 95% confidence interval [CI]: 1.22–1.73), a previous history of TB (aOR = 1.50, 95% CI: 1.09–2.07), and being employed/self-employed (aOR = 1.21, 95% CI: 1.02–1.43) were independent influential factors for LTFU before the treatment regimen. The protective factors were had ever been hospitalized for TB (aOR = 0.22, 95% CI: 0.18–0.28) and admission to emergency department (aOR = 0.38, 95% CI: 0.31–0.47) (Table 3).

The independent risk factors for LTFU during the treatment were negative AFB smear/GeneXpert/culture results (aOR = 1.75, 95% CI: 1.58–1.94), chronic hepatitis or cirrhosis (aOR = 1.24, 95% CI: 1.02–1.51), the occurrence of adverse drug reactions (aOR = 1.26, 95% CI: 1.03–1.52) and being employed/self-employed (aOR = 1.15, 95% CI: 1.04–1.27). Patients who were consulted in the emergency department (aOR = 0.40, 95% CI: 0.35–0.44) had a lower incidence of LTFU compared with those consulted in an outpatient clinic. Patients with medical insurance (aOR = 0.76, 95% CI: 0.69–0.85), the provision of an alternative contact (aOR = 0.82, 95% CI: 0.72–0.94), and hospitalization (aOR = 0.19, 95% CI: 0.17–0.22) were protective factors for LTFU after treatment initiation (Table 3).

## Discussion

This is the first large cohort study to analyze the potential predictors of LTFU in patients with TB in China according to the WHO’s definition of LTFU, which divides lost cases into two phases: before and after the initiation of treatment. In our study, the incidence of LTFU in all patients with TB was 12.55% (3,046/24,265), which is lower than other high TB prevalence countries such as South Africa (20%), India (19%), and Angola (28%) (18–20). However, China, as a nation with a high incident burden, has remaining obstacles to completing the regimen successfully, and it is especially hindered by patients who are LTFU. Identifying the precise risk factors and the incident time of LTFU would benefit the optimization of follow-up protocols and ultimately limit LTFU in patients with TB.

Our findings, as further research, demonstrated that patients with a TB history were more likely not to receive treatment, and some were lost even before treatment, with previous studies suggesting that such patients were more likely to be non-adherent to anti-TB treatment (21). Among patients with a previous TB history, the cases who were consulted in outpatient clinics had a higher LTFU incidence before regimen initiation than after. This indicates that re-treated patients with TB should be given more health education and follow-up reminders when they are consulted in an outpatient clinic, especially before treatment starts. In addition, the higher frequency of LTFU in re-treated patients may be attributed to psychological distress, lack of social support, stigma, and negative attitudes toward treatment in other studies (17, 22, 23). Patients who have a previous history of TB were also one of the highest risk factors for DR TB (24). Therefore, it is essential to strengthen the management of re-treated TB patients before initiating treatment in order to reduce the incidence of drug-resistant TB cases, deaths, and community transmission. An effective approach to achieve this objective is to provide a thorough evaluation for these patients before commencing treatment, which includes drug susceptibility testing (DST) to identify any drug-resistant strains. This measure ensures that patients receive the appropriate medication regimen from the outset, reducing the likelihood of treatment failure and the emergence of drug resistance. Moreover, close monitoring of re-treated patients throughout their treatment and ensuring their adherence to medication regimens can also prevent treatment failure and the spread of TB.

In our study, the majority of patients who were LTFU were lost during the initial phase of treatment (i.e., the first 3 months). Our results were broadly similar to those of studies conducted in other nations, which helps to validate the current ongoing pattern of LTFU during TB treatment (12, 25). Intensive observation during frequent contact between healthcare workers and patients *via* direct observation and home visits can improve adherence (26). Furthermore, targeted and personalized interventions based on the occurrence and time characteristics of LTFU may help to reduce the loss, although this strategy needs to be substantiated by further research. Our study provides an evidence base for understanding the characteristics of LTFU during TB treatment and identifying potential interventions to reduce losses.

We found that individuals who had ever been hospitalized for TB more likely than other patients not to be lost in the future. In the hospital, patients receive additional health education, which helps to enhance adherence (27), and full-time medical care is provided to patients with TB in treatment units until they meet the discharge standards of the hospital. This period of gradual improvement in conditions strengthens confidence in ongoing treatment (28).

Being admitted to hospital on an emergency basis was also a protective factor for LTFU, which was previously unidentified. Emergency cases usually have acute symptoms or complex illnesses, such as high fever, chest pain, coughing up blood, and difficulty breathing. Because patients may face serious health risks, this prompts them to seek medical help. Patients usually seek medical help immediately, and consult with professional doctors for advice and treatment, in order to alleviate symptoms and prevent the further development of the disease. Adherence to treatment was enhanced in those who received more chances to communicate with healthcare providers (28).

Our results revealed that co-morbidity with chronic hepatitis or cirrhosis was a risk factor for LTFU during anti-TB treatment, but it was not affected before treatment initiation. This can be explained by the fact that patients with chronic liver disease are more prone to hepatotoxicity during anti-TB regimens (29), which results in patients discontinuing their treatment to prevent further liver damage. Furthermore, we found that patients who experienced adverse reactions had a higher risk of LTFU after the initiation of treatment. Serious drug side effects cause patients to discontinue their treatment. Frequently changed regimens caused by adverse reactions may result in people losing patience with their treatment, resulting in treatment withdrawal and follow-up interruption (8, 13). Therefore, it is necessary to strengthen the training of medical staff, improve their ability to monitor and manage adverse drug reactions. At the same time, a sound reporting and management mechanism for adverse drug reactions should be established to help patients overcome treatment difficulties and improve treatment compliance (8, 13).

The differences in LTFU levels in our cohort were compared in terms of the provision of an alternative contact person while registering on the TBIMS. It is worth noting that the registration of an alternative contact is an essential protective component of LFTU after anti-TB initiation. It is obvious that an alternative contact, generally the patient’s family, can assist the healthcare provider with communication, organizing follow-up appointments, and ensuring treatment adherence. The careful documentation of patient information during therapy may be valuable for patient management and treatment decision making as well as for providing additional data to support local TB elimination strategies (30).

Our analysis revealed that patients with negative laboratory test results had a higher likelihood of LTFU. This finding consisted with a study conducted in France that found that patients with negative smears were less likely to start anti-TB treatment (31). This highlights the need for healthcare providers to closely monitor patients with negative laboratory test results to prevent them from being lost to follow-up and ensure timely initiation of treatment. Our study found that medical insurance is associated with a lower risk of LTFU in tuberculosis patients. This supports previous research which demonstrated that patients without medical insurance are more likely to experience poor treatment outcomes (32). Medical insurance in China refers to a system where individuals pay a fee to access healthcare services, and the costs are shared by the insurance provider and the individual. The absence of medical insurance means higher medical costs. The “Law of the People’s Republic of China on the Prevention and Treatment of Tuberculosis” encourages and supports insurance institutions and medical facilities to provide services for insured individuals, including reimbursement of expenses and designated medical treatment (33). As an infectious disease associated with poverty, effective TB management interventions should target the alleviation of the financial burden faced by patients with TB (33). Furthermore, being employed or self-employed was an independent risk factor for LTFU. This finding was different from the previous results demonstrated that being unemployed was associated with LTFU (34, 35). We hypothesize that this result was due to the majority of employed or self-employed cases being exposed to social and work stresses, which can sustain negative impacts and contribute to a high risk of LTFU (36).

In addition to the factors previously mentioned that are associated with loss to follow-up, there are other factors related to low adherence that have been identified in the literature. For instance, a study conducted in China found that delayed healthcare seeking was linked to low treatment adherence among patients with tuberculosis (36). Furthermore, the choice of TB treatment regimen may impact treatment adherence (37). Additionally, inadequate drug monitoring can lead to poor adherence, as insufficient or excessive drug concentrations can affect treatment efficacy and the occurrence of adverse drug reactions (38). Finally, non-adherence to medication is another important factor contributing to poor treatment adherence. Specifically, patients may forget to take their medication, lack a fixed time and place for taking medication, or stop taking it, all of which can result in non-adherence (39).

Our retrospective study has additional limitations. First, some factors that have been previously reported affecting LTFU were inaccessible in our dataset. These factors include distance to treatment units, patient knowledge of tuberculosis, attitude toward treatment, and treatment beliefs ^[43, 44]^. Second, this study only assessed reginal database in Shenzhen, a city of immigrants and well-developed city. Therefore, the result is less representative for other areas of China. However, our findings have provided insight on the management of LTFU in TB treatment, and this may benefit TB surveillance and control in China.

## Conclusion

In summary, the incidence of LTFU in TB patients in my country remains relatively high, posing a severe challenge to disease control. Most LTFU occur in the early stages of antituberculosis treatment. The majority of LTFU cases occur during the early stages of antituberculosis treatment. Strategies to enhance treatment adherence and reduce LTFU include implementing rigorous monitoring and management of adverse reactions, along with frequent follow-up to supervise and encourage patients to adhere to their treatment regimen. Additionally, providing comprehensive information about TB and effective treatment options, and addressing patients’ concerns and doubts regarding TB treatment, can improve patients’ awareness and attitudes towards TB care. These measures can help promote patient engagement and ultimately improve their treatment compliance.

## Data availability statement

The data supporting this research conclusion can be obtained by contacting the authors (YJ and JC) at h2362120381@163com and 13823139640@163.com.

## Ethics statement

Written informed consent was obtained from the individual(s), and minor(s)’ legal guardian/next of kin, for the publication of any potentially identifiable images or data included in this article.

## Author contributions

JC and HL contributed to the conceptualization of the article and conceived this study. YJ wrote the original draft and founding acquisition. MY, LL, and ZL analyzed the data. XL, SL, and PZ reviewed the manuscript. QX and ML collected the data. HL contributed to the supervision and validation of the article. All authors contributed to the article and approved the submitted version.

## Funding

This work was supported by Shenzhen High-level Hospital Construction Fund (G2022006).

## Conflict of interest

The authors declare that the research was conducted in the absence of any commercial or financial relationships that could be construed as a potential conflict of interest.

## Publisher’s note

All claims expressed in this article are solely those of the authors and do not necessarily represent those of their affiliated organizations, or those of the publisher, the editors and the reviewers. Any product that may be evaluated in this article, or claim that may be made by its manufacturer, is not guaranteed or endorsed by the publisher.
